# Nocturnal insomnia symptoms and stress-induced cognitive intrusions in risk for depression: A 2-year prospective study

**DOI:** 10.1371/journal.pone.0192088

**Published:** 2018-02-13

**Authors:** David A. Kalmbach, Vivek Pillai, Christopher L. Drake

**Affiliations:** 1 Department of Psychiatry, University of Michigan Medical School, Ann Arbor, Michigan, United States of America; 2 Sleep Disorders and Research Center, Henry Ford Hospital, Detroit, Michigan, United States of America; Neurocenter of Southern Switzerland, SWITZERLAND

## Abstract

Nearly half of US adults endorse insomnia symptoms. Sleep problems increase risk for depression during stress, but the mechanisms are unclear. During high stress, individuals having difficulty falling or staying asleep may be vulnerable to cognitive intrusions after stressful events, given that the inability to sleep creates a period of unstructured and socially isolated time in bed. We investigated the unique and combined effects of insomnia symptoms and stress-induced cognitive intrusions on risk for incident depression. 1126 non-depressed US adults with no history of DSM-5 insomnia disorder completed 3 annual web-based surveys on sleep, stress, and depression. We examined whether nocturnal insomnia symptoms and stress-induced cognitive intrusions predicted depression 1y and 2y later. Finally, we compared depression-risk across four groups: non-perseverators with good sleep, non-perseverators with insomnia symptoms, perseverators with good sleep, and perseverators with insomnia symptoms. Insomnia symptoms (β = .10–.13, p < .001) and cognitive intrusions (β = .19–.20, p < .001) predicted depression severity 1y and 2y later. Depression incidence across 2 years was 6.2%. Perseverators with insomnia had the highest rates of depression (13.0%), whereas good sleeping non-perseverators had the lowest rates (3.3%, Relative Risk = 3.94). Perseverators with sleep latency >30 m reported greater depression than good sleeping perseverators (t = 2.09, p < .04). Cognitive intrusions following stress creates a depressogenic mindset, and nocturnal wakefulness may augment the effects of cognitive arousal on depression development. Poor sleepers may be especially vulnerable to cognitive intrusions when having difficulty initiating sleep. As treatable behaviors, nighttime wakefulness and cognitive arousal may be targeted to reduce risk for depression in poor sleepers.

## Introduction

Exposure to stress is widely recognized as a common precipitant of depression [1, 2], and poor sleepers are twice as likely to become depressed during chronic stress than good sleepers [3]. More so than the exposure itself, the manner in which individuals react to stressful events dictates the emotional and somnological fallout [4–11]. Cognitive arousal and intrusions in response to stressors precludes adaptive emotion regulation, thereby giving rise to and prolonging negative affect states [5, 10, 12–18]. Individuals having difficulty falling or staying asleep may be especially vulnerable to intrusive thoughts given that the inability to sleep creates a period of unstructured time in bed at night marked by social isolation and limited behavioral distractions: a veritable tabula rasa for perseverating on one’s trials and tribulations. Indeed, presleep cognitive arousal is associated with poor sleep quality and is often a key ingredient in insomnia [19–31] (please refer to excellent reviews on sleep and rumination by Espie et al. [32], Harvey [11], Pillai and Drake [20], and Riemann et al. [29] for in-depth analysis). Importantly, cognitive arousal, as a transdiagnostic cognitive process, has been proposed as a key ingredient linking sleep problems and depression [11, 20, 33–35].

Although clinical insomnia disorder is a well-established risk factor for depression [36, 37], individuals with subclinical insomnia symptoms are also at elevated depression-risk [38], particularly during high stress [3]. Given alarming estimates that 30–48% of US adults complain of difficulty falling or staying asleep [39], a substantial portion of the US population may be especially vulnerable to emotionally corrosive nocturnal cognitive arousal when stressed. That is, as difficulty sleeping creates periods of nocturnal wakefulness susceptible to intrusive thoughts, these otherwise healthy individuals are likely at higher risk for developing depression during stress than good sleepers or those not predisposed to cognitive arousal. However, we know of no studies to-date that have prospectively examined the independent and combined effects of nocturnal insomnia symptoms and stress-induced cognitive arousal on incident depression.

To explore the independent and combined effects of nocturnal insomnia symptoms and stress-induced cognitive arousal on risk for developing depression, we collected three annual assessments of sleep, mood, and stress across two years from a community sample of never-depressed US adults with no history of DSM-5 insomnia disorder. We predicted that longer nighttime wakefulness (sleep latency + wake after sleep onset) and cognitive intrusions in response stressful life events would correspond to self-reported depression severity one and two years later. Next, we compared risk for screening positive for clinical depression across the following groups: 1. subjects with low cognitive intrusions and no insomnia symptoms (i.e., non-perseverators with good sleep), 2. non-perseverators with nocturnal insomnia, 3. perseverators with good sleep, and 4. perseverators with nocturnal insomnia. Consistent with the proposal that difficulties falling/staying asleep and cognitive arousal have synergistic effects, we predicted that subjects with insomnia and high cognitive intrusions would report the greatest depression.

## Methods

### Subjection recruitment and screening

Data were collected via web-based surveys from a large community sample in Southeastern Michigan as part of the prospective NIMH-funded *Evolutions of Pathways to Insomnia Cohort* (EPIC) Study. Recruitment, eligibility, and demographic information have been reported in great detail in several other reports [40–43]. In short, 36,002 individuals randomly selected from an HMO database were invited to participate, with 7,608 completing an online eligibility survey. After excluding those who met criteria for DSM-5 [44] insomnia disorder or major depressive disorder (MDD), as well as inconsistent or indiscriminate responders, 4,869 individuals were eligible to participate. We collected baseline data on 2,892 of these individuals.

For the present study, we implemented additional inclusionary criteria: age ≥ 18 years (excluded n = 1), no missing data on key baseline variables (gender [excluded n = 2], insomnia symptoms [excluded n = 12], cognitive intrusions [excluded n = 595]), experienced at least one major life stressor in the past year (excluded n = 518, note: stress-related cognitive intrusion data were only collected from subjects who endorsed exposure to stress), negative depression screen on self-report survey (to ensure a non-depressed sample, excluded n = 227, see *Measurements* below), in the top or bottom tercile on stress-induced cognitive arousal (to create groups of perseverators and non-perseverators, excluding n = 1373), and no extreme outliers in reported nocturnal wakefulness (sleep latency + wake after sleep onset < 90 minutes, per interquartile rule, excluding 12). As a result, our final sample consisted of 1,126 individuals (NB: when summed, the above exclusion numbers exceed the number of subjects excluded due to some excluded subjects violating multiple exclusion categories); see Table 1 for full characteristics.

**Table 1:** Sample characteristics.

	All subjects	Non-Perseverators		Perseverators		
		Good Sleep	Insomnia Symptoms	Good Sleep	Insomnia Symptoms	
Sample size	1126	520	122; 16.44%	350	134; 27.69%	
Age	46.80±13.29	47.68±13.26	45.75±13.64	46.80±13.24	44.37±13.01	F = 2.51, p = .06
Gender (Women)	651; 57.8%	241; 46.3%^c^^,^^d^	69; 56.6%^d^	241; 68.9%^a^	100; 74.6%^a^^,^^b^	χ2 = 61.50, p < .001
Race						χ2 = 54.94, p < .001
White	752; 66.8%	370; 71.2%^b^^,^^d^	67; 54.9%^a^^,^^d^	241; 68.9%^b^^,^^d^	74; 55.2%^a^^,^^c^	
Black	245; 21.8%	84; 16.2%^b^^,^^d^	40; 32.8%^a^	75; 21.4%^b^^,^^d^	46; 34.3%^a^^,^^c^	
Asian	53; 4.7%	33; 6.3%	8; 6.6%	10; 2.9%	2; 1.5%	
Hispanic or Latinx	24; 2.1%	10; 1.9%	3; 2.5%	9; 2.6%	2; 1.5%	
Middle Eastern or Indian	38; 3.4%	15 2.9%	3; 2.5%	13; 3.7%	7; 5.2%	
Other	13; 1.2%	8; 1.5%	1; 0.8%	2; 0.6%	3; 2.2%	
Major Life Events	2.43±1.59	2.13±1.38^c^^,^^d^	2.26±1.57^d^	2.67±1.60^a^^,^^d^	3.09±2.04^a^^,^^b^^,^^c^	F = 17.50, p < .001
IES-I	14.03±5.78	9.29±1.72^c^^,^^d^	9.51±1.82^c^^,^^d^	20.14±2.19^a^^,^^b^	20.59±2.63^a^^,^^b^	F = 2720.75, p < .001
SOL	22.83±16.61	15.18±7.99^b^^,^^c^^,^^d^	42.75±19.23^a^^,^^c^^,^^d^	17.74±8.77^a^^,^^b^^,^^d^	47.69±16.23^a^^,^^b^^,^^c^	F = 456.49, p < .001
WASO	13.42±12.64	9.19±7.26^b^^,^^d^	25.78±19.72^a^^,^^c^	11.19±7.74^b^^,^^d^	24.40±17.06^a^^,^^c^	F = 127.94, p < .001

Notes:

^a^ = group mean value or sample proportion differs from *non-perseverators with no insomnia* group mean value.

^b^ = group mean value or sample proportion differs from *non-perseverators with insomnia* group mean value.

^c^ = group mean value or sample proportion differs from *perseverators with no insomnia* group mean value.

^d^ = group mean value or sample proportion differs from *perseverators with insomnia* group mean value.

### Procedure

All study protocols were approved by the Henry Ford Hospital institutional review board. Individuals were required to provide informed consent prior to participating. The setting of this study was a web-based epidemiological survey, which were collected in annual waves across two years. Baseline data were collected after subjects met eligibility requirements using web-delivered questionnaires. One month prior to each annual follow-up (at Years 1 and 2), subjects received email reminders and invitations to complete surveys. Each assessment took approximately 30 minutes to complete.

### Measures

#### Insomnia symptoms

At baseline, subjects were asked to estimate their sleep onset latency (SOL: ‘On average, including weekdays and weekends, how long does it take you to fall asleep in the past month?’) and wake after sleep onset (WASO: ‘On average, how long does it take you to fall back asleep after waking up during the past month?’). Individuals reporting SOL and/or WASO > 30 minutes were classified as having insomnia symptoms, per established quantitative cutoffs [45]. In addition, we summed the estimates of SOL and WASO for each subject to create a *nocturnal wakefulness* variable representing the duration of time individuals are awake during the intended sleep period.

#### Stress exposure and cognitive arousal

Stress exposure was reported at baseline using the Social Readjustment Rating Scale (SRRS-R) [46, 47], an inventory of 52 stressful life events commonly reported by US adults (e.g., divorce, change in residence, loss of employment). Subjects reported the number of life events experience over the past year. To assess stress-induced cognitive intrusions and arousal, we administered the eight-item cognitive intrusion subscale of the revised impact of events scale (IES-I) in response to each endorsed stressor [48]. The IES-I measures the presence and pervasiveness of recurrent, intrusive thoughts and perseverations (e.g., “I thought about it when I didn’t mean to”, “other things kept making me think about it”) in response to a stressor on a four-point Likert-type scale. Scores here represent subjects’ mean IES-I ratings in response to major life events, with scores ranging from 7 to 28 (14.88±4.89). To split the sample into subjects with high levels of cognitive intrusions (henceforth, ‘perseverators’) and low levels of cognitive intrusions (‘non-perseverators’), we retained those who scored in the upper and lower terciles, and excluded subjects who scored in the middle tercile.

#### Depression

Depression symptom severity and status served as our outcomes of interest, and were assessed using the 16-item version of the Quick Inventory of Depressive Symptomatology (QIDS) [49]. The QIDS has a total score range from 0 to 27 with higher scores indicating greater depression severity. To reduce collinearity with insomnia symptoms, the sleep disturbance items of the QIDS were excluded. Traditionally, total scores of 11 or higher indicate moderate depression symptoms. To account for the elimination of the sleep subscale, we used a cutoff of QIDS ≥ 10 in the present study. The range of scores on the QIDS-16 is 0 to 27. Thus, a cutoff of ≥ 11 corresponds to the 40.74th percentile of the scale’s total 27 points. After removing the sleep subscale, the maximum score possible is 24. The 40.74th percentile then corresponds to a score of 9.78, which we rounded to ≥10 to create our adjusted cutoff for moderate depression. Subjects with QIDS scores < 10 at baseline were included in the study (to ensure a non-depressed sample), whereas subjects who reported QIDS scores ≥ 10 at 1- and 2-y follow-up were categorized as depressed.

### Analysis

We used SPSS 24 to conduct all analyses. Depression symptoms and status at 1y and 2y after baseline served as our outcomes for linear and logistic regression models. In comparing risk for developing depression, we used dummy coded logistic regression to compare depression-risk across four groups based on cognitive arousal and insomnia symptoms: (1) non-perseverators without insomnia (reference group); (2) non-perseverators with insomnia; (3) perseverators without insomnia; and (4) perseverators with insomnia. In all regression models, we controlled for the potentially confounding effects of age, gender, and stress exposure at baseline.

## Results

### Sample characteristics

At baseline, 22.7% (n = 256/1126) endorsed insomnia symptoms (SOL and/or WASO > 30 min). Among those with baseline insomnia symptoms, 53.5% (n = 141/255) endorsed insomnia symptoms again a year after baseline assessment. Individuals with high cognitive arousal in response to stressful events were more likely than non-perseverators to have insomnia symptoms (27.69% vs 19.00%, Relative Risk [RR] = 1.46; logistic regression: b = .49, Odds-Ratio [OR] = 1.63, 95% CI = 1.23–2.16). We then compared demographics across four groups: (1) non-perseverators with good sleep, (2) non-perseverators with insomnia, (3) perseverators with good sleep, and (4) perseverators with insomnia; see Table 1 for full sample characteristics. Age did not differ across groups. Women were most over-represented among perseverators with insomnia (74.6%) and most under-represented among non-perseverators without insomnia (46.3%; RR = 1.61). Non-Hispanic whites were over-represented among good sleepers, whereas blacks were over-represented among those with insomnia, irrespective of stress-response style.

### Risk for depression development within one year

Depression incidence was 4.1% (n = 46/1126) a year after baseline. Using linear regression, we predicted depression symptoms at 1y as estimated by baseline nocturnal wakefulness (SOL+WASO) and cognitive arousal, while controlling for age, gender, and stress exposure in the year preceding baseline (Table 2, Model 1). Analyses revealed that greater nocturnal wakefulness (β = .10, p < .001) and stress-induced cognitive arousal (β = .19, p < .001) were associated with greater depression symptoms a year later, and that these effects were independent of age (b = -.02, p < .01), female gender (b = .36, p = .03), and stress exposure (b = .22, p < .001).

**Table 2:** Insomnia and cognitive arousal predicting depression one and two years later.

	b	ES	95% CI	sig.
Model 1: *Depressive Symptoms at 1y*		*β*		
Age	-.02	-.08	−.03–−.01	< .01
Female Gender	.36	.06	.04–.68	.03
Major Life Events	.22	.13	.12–.32	< .001
Nocturnal Wakefulness	.01	.10	.01–.02	< .001
Cognitive Arousal	.09	.19	.06–.12	< .001
Model 2: *Depression Status at 1y*		*OR*		
Age	-.02	.98	.96–1.01	.13
Female Gender	.80	2.22	1.06–4.64	.03
Major Life Events	.26	1.30	1.12–1.50	< .001
Non-perseverators with insomnia	.24	1.27	.40–4.06	.69
Perseverators with good sleep	.61	1.85	.87–3.92	.11
Perseverators with insomnia	.82	2.27	.94–5.49	.07
Model 3: *Depressive Symptoms at 2y*		*β*		
Age	−.00	−.01	−.02–.01	.69
Female Gender	−.02	−.12	−.39–.34	.91
Major Life Events	.14	.08	.02–.25	.02
Nocturnal Wakefulness	.02	.13	.01–.02	< .001
Cognitive Arousal	.09	.20	.06–.12	< .001
Model 4: *Depression Status at 2y*		*OR*		
Age	-.02	.99	.96–1.01	.27
Female Gender	-.32	.73	.33–1.60	.55
Major Life Events	.07	1.07	.85–1.35	.55
Non-perseverators with insomnia	-.61	.54	.07–4.43	.57
Perseverators with good sleep	.87	2.38	.94–5.98	.07
Perseverators with insomnia	1.23	3.43	1.15–10.21	.03
Model 5: *Depression Status at 1y or 2y*		*OR*		
Age	-.01	.99	.97–1.01	.28
Female Gender	.11	1.12	.59–2.12	.74
Major Life Events	.18	1.19	1.01–1.41	.04
Non-erseverators with insomnia	.04	1.04	.28–3.80	.96
Perseverators with good sleep	.87	2.38	1.13–5.00	.02
Perseverators with insomnia	1.28	3.61	1.54–8.48	< .01

Notes: Models 2, 4, and 5 employ dummy coding to compare groups of perseverators and poor sleepers to the reference group consistent of non-perseverating good sleepers. *Depressive Symptoms* measured by the Quick Inventory of Depressive Symptomatology (QIDS) 16-item version. *Depression Status* also measured by the QIDS with scores < 10 conferring a negative screen, and scores ≥ 10 indicating a positive depression screen. 1y = one year after baseline. 2y = two years after baseline. b = beta, i.e., unstandardized regression coefficient. ES = effect size, referring to β (standardized regression coefficients) for linear regression models and OR (Odds-Ratio) for logistic regression, as denoted in the column. CI = confidence interval, which represents the 95% CI around b (linear models) and OR (logistic models). sig. = p-value indicating statistical significance.

Chi-square analysis indicated that depression incidence rates at 1y significantly differed across the four groups as defined by baseline cognitive arousal and sleep classifications (χ^2^ = 11.82, p < .01). Data presented in Table 3 show depression rates to be highest among perseverators with insomnia (8.2%) and lowest for good sleeping non-perseverators (2.3%). We then conducted dummy coded logistic regression to compare depression-risk among the groups (reference group: non-perseverators with good sleep; see Table 2, Model 2). Elevated depression-risk among perseverators with insomnia trended toward significance (OR = 2.27, p = .07), whereas the other groups of perseverators and poor sleepers did not differ from the good sleeping, non-perseverating reference group.

**Table 3:** Rates of depression based on cognitive arousal and insomnia symptoms.

	Depression Status				
	No	Yes	% Depressed	RR	χ^2^, sig.
*Perseverative style & sleep*	At 1y follow-up				χ^2^ = 11.82, p < .01
Non-perseverators, good sleep	508	12	2.3%	--	
Non-perseverators, insomnia	118	4	3.3%	1.43	
Perseverators, good sleep	331	19	5.4%	2.35	
Perseverators, insomnia	123	11	8.2%	3.57	
	At 2y follow-up				χ^2^ = 8.10, p = .04
Non-perseverators, good sleep	357	8	2.2%	--	
Non-perseverators, insomnia	79	1	1.3%	.59	
Perseverators, good sleep	249	13	5.0%	2.27	
Perseverators, insomnia	93	7	7.0%	3.18	
	At 1y or 2y follow-up				χ^2^ = 16.29, p < .001
Non-perseverators, good sleep	353	12	3.3%	--	
Non-perseverators, insomnia	77	3	3.8%	1.15	
Perseverators, good sleep	240	22	8.4%	2.55	
Perseverators, insomnia	87	13	13.0%	3.94	

Notes: *Depression Status* also measured by the QIDS with scores < 10 conferring a negative screen, and scores ≥ 10 indicating a positive depression screen. 1y = one year after baseline. 2y = two years after baseline. RR = relative risk, here all RRs are compared to the *non-perseverators with good sleep* group. χ^2^ = chi-square value for comparing depression rates. sig. = p-value indicating statistical significance.

To determine whether the effects of baseline nocturnal wakefulness and stress-focused cognitive arousal on depression are independent of changes in insomnia symptoms and stressful life events between baseline and 1y follow-up, we re-ran Model 1 (Table 2) controlling for these factors. While both increases in nocturnal wakefulness (i.e., Nocturnal wakefulness_1y-0y_: b = .003, p = .01) and exposure to new stressful events (b = .36, p < .001) significantly predicted greater depressive symptoms at 1y follow-up, the effects of baseline nocturnal wakefulness (b = .01, p = .001) and cognitive arousal (b = .08, p < .001) on depressive symptoms remained unchanged.

To evaluate whether insomnia persistence predicted depression severity, we then conducted a *posthoc* one-way ANOVA comparing three groups based on insomnia symptoms: 1. good sleepers (no insomnia endorsement at 0y or 1y), 2. subjects with acute insomnia symptoms (endorsed insomnia symptoms at 0y or 1y only), and 3. subjects with persistent insomnia symptoms (endorsed insomnia symptoms at 0y and 1y), followed by both Bonferroni and LSD group comparisons. The overall ANOVA model revealed that depressive symptoms at 1y follow-up differed across good sleepers (M±SD: 3.37±2.54), subjects with acute insomnia symptoms (4.05±3.14), and those with persistent insomnia (4.65±2.97), F[2,1118] = 16.04, p < .001. LSD comparisons suggested that subjects with persistent insomnia reported greater depression than subjects with acute insomnia (Mean difference = .60, p = .04) and good sleepers (Mean difference = 1.28, p < .001), and that subjects with acute insomnia also reported more depression than good sleepers (Mean difference = .68, p = .001). Bonferroni comparisons, which are more conservative than LSD comparisons, suggested that both the acute insomnia (p < .01) and persistent insomnia (p < .001) groups endorsed more depression than good sleepers, but that they did not differ between one another (p = .12).

### Risk for depression after two years

Rates of depression were 3.6% (n = 29/807) two years after baseline. Consistent with our first linear regression model, nocturnal wakefulness (β = .13, p < .001) and stress-induced cognitive arousal (β = .20, p < .001) predicted depression symptoms two years post-baseline, the effects of which were again independent of stress exposure (b = .14, p = .02; Table 2, Model 3). Age and gender were not significant in this model.

Groups again differed in rates of depression (χ^2^ = 8.10, p = .04, Table 3) such that perseverators with insomnia had the highest rates of depression (7.0%) and good sleeping non-perseverators had the lowest (2.2%). A dummy coded logistic regression model estimating depression status showed that perseverators with insomnia were at more than three-folds greater odds for depression at 2y than non-perseverating good sleepers (OR = 3.43, p = .03; Table 2 Model 4). Notably, elevated depression-risk approached significance for perseverators without insomnia (OR = 2.38, p = .07).

### Depression incidence within two years

We then collapsed across 1y and 2y follow-up assessments to replicate the above findings for cases of incident depression across two years. The two-year incidence of depression was 6.2% (50/807). Expectedly, chi square analysis showed significant group differences in depression rates (χ^2^ = 16.29, p < .001, Table 3) with perseverating poor sleepers reporting the highest rates of depression incidence (13.0%) and non-perseverating good sleepers having the lowest rates (3.3%). Dummy coded logistic regression showed that perseverators with insomnia (OR = 3.61, p < .01) and without insomnia (OR = 2.38, p = .02) were at greater risk for depression than the good sleeping non-perseverators (Table 2, Model 5), with perseverating poor sleepers having 1.55 times the risk for depression than perseverators with good sleep (13.0% vs 8.4%, Table 3). A *posthoc* group comparison showed that perseverating poor sleepers reported higher average depression levels across 1y and 2y follow-ups than non-perseverating good sleepers (QIDS_(Y1+Y2)/2_: 4.92±2.22 vs 4.39±2.22, t = 2.30, p = .02, Cohen’s d = .24).

### Pathogenicity of prolonged sleep latency vs poor sleep maintenance

Finally, we sought to identify which component(s) of insomnia symptoms (SOL and/or WASO) contributed to the difference in depression among perseverators with vs without insomnia symptoms. First, we found that perseverators with significant sleep latency (>30 minutes) reported greater severity of depression than perseverators without difficulty falling asleep (≤30 minutes) across the two follow-up assessments (QIDS_(Y1+Y2)/2_: 4.94±2.22 vs 4.43±2.22, t = 2.09, p < .04, Cohen’s d = .23). In contrast, depression symptoms across two years did not differ between perseverators with vs without prolonged WASO (QIDS_(Y1+Y2)/2_: 4.97±2.27 vs 4.53±2.23, t = .49, p = .62).

## Discussion

The inability to adaptively cope with stressful life events creates a depressogenic mindset, and evidence from the present study support synergistic effects of cognitive arousal and nocturnal wakefulness on depression development. In a sample of 1,126 never-depressed US adults with no history of insomnia disorder, we observed that difficulty falling and staying asleep and cognitive intrusions about stressful events augured incident depression one and two years later. The consequence of which resulted in cognitively aroused poor sleepers were 3–4 times more likely to develop depression than good sleepers without cognitive arousal. Critically, perseverating poor sleepers were at higher risk for depression than perseverators who slept well.

These findings suggest that individuals experiencing sleep disturbance are at elevated risk for MDD—a disorder flush with unmoored ruminations on perceived immutable stressors and personal defects—when faced with major life stressors, as their nocturnal wakefulness provides a breeding ground for intrusive thoughts. Indeed, nocturnal cognitive arousal is well-documented in insomnia [11, 26, 29, 32, 50, 51]. Importantly, however, insomnia research has almost exclusively focused on cognitive arousal and reactivity as the *cause* of nocturnal wakefulness, rather than its *consequence*. Although nocturnal rumination can impede sleep onset [30, 52, 53], the failure to fall/remain asleep can also trigger cognitive arousal, thus creating a positive feedback cycle. The quiet, dark periods of nocturnal wakefulness in poor sleepers are marked by increased social isolation on account of being awake when others are asleep [54]. Further, this period is unstructured with no *a priori* behavioral directives [55]. Thus, nocturnal wakefulness provides a vulnerable period for the mind to wander and race in poor sleepers, thereby extending cognitive intrusions into the night and further increasing likelihood of depression [35, 56]. By comparison, good sleepers get an adaptive reprieve from stress-focused perseverations at bedtime, which may mitigate some of the potential effects of cognitive arousal and ruminative coping on mood.

Indeed, our findings showed that worsening of insomnia symptoms and persistent insomnia predicted greater depression. The inability to fall asleep at night can itself trigger negative schema, such as helplessness and lack of control, that provide additional content for cognitive arousal [57]. And analysis of presleep cognitions show that insomniacs are more likely to engage in nocturnal perseveration and for longer durations than good sleepers [51], with negatively-valenced thoughts likely to occur while trying to sleep [58]. Given that sleep onset difficulties are linked to negative perseverative thinking [30, 52, 53] and are often prodromal and presage more severe and debilitating forms of insomnia [7, 59], it is perhaps unsurprising that sleep latency appears to be a key insomnia ingredient contributing to depression in perseverators in the current study. When the cortex is a flurry of activity during the solitary and distraction-free presleep period, a toxic nocturnal environment for both mood and sleep is created.

Early identification of individuals with disrupted sleep and exaggerated cognitive reactivity to stress may improve depression prevention. Fortunately, insomnia symptoms and cognitive intrusions are treatable targets. Personalized interventions focusing on improving sleep, particularly its initiation, may reduce the risk for depression for cognitively aroused poor sleepers. For those with subclinical insomnia, digital delivery of cognitive behavioral therapy for insomnia (dCBTI) [60–62] may be a cost-effective and easily accessible intervention option to potentially reduce risk for MDD and insomnia disorder. Sleep-focused prevention efforts may be especially appropriate for black Americans who were over-represented among the poor sleepers in this study, in addition to other reports suggesting that sleep problems contribute to the greater disease burden for racial minorities [41, 63–65]. Alternatively, or perhaps better yet adjunctively, individuals with high cognitive reactivity to stress may decrease their risk for depression through training in mindfulness [66, 67] or other decentering skills, which can reduce overall cognitive intrusions and may be deployed during nocturnal wake bouts. Skills to alleviate cognitive arousal may also reduce insomnia-risk, as perseverating on sleep problems [22, 68] and stressors [6, 28] contributes to insomnia disorder development. Those with a prior history of insomnia disorder or psychiatric illness would be likely best served to consult their primary health provider or a specialist in sleep or mental health services for early intervention.

### Limitations

The present study should be interpreted in light of certain limitations. Though the QIDS has demonstrated excellent diagnostic utility as a screening tool [49], clinician interviews are necessary for proper diagnosis of psychiatric disorders. Further, while subjective sleep ratings are shown to correspond to objective sleep measures [69, 70], the gold standard of sleep assessment involves the combination of subjective ratings and objective indicators (e.g., actigraphy, polysomnography). And although the quantitative cutoffs for self-reported sleep latency and wake after sleep onset we used in this study have been empirically validated for nocturnal insomnia symptoms [45], these data remain limited by their subjective and retrospective nature. It is also worth noting that we did not explicitly assess early morning awakenings in subjects, and thus likely did not capture the late/terminal insomnia phenotype. It is possible that difficulty falling back asleep late in the sleep period may be conducive to perseverating on stressful events or even the day’s oncoming trials and tribulations. In addition, our study focused largely on nocturnal processes and symptoms associated with insomnia, thus future research is needed to account for the effects of diurnal insomnia symptoms. Lastly, though our data support unique and combined effects of cognitive arousal and insomnia on future depression-risk, due to the observational study design, we cannot establish causal mechanisms. As described above, prior research supports a bidirectional relationship between nocturnal cognitive arousal and insomnia symptoms, and future prospective analysis is needed to evaluate how these inner-machinations affect long term outcomes.

### Conclusions

The present study offers strong evidence of the insalubrious environment created by nocturnal insomnia and difficulty corralling one’s thoughts on life stress. Investigations are needed to explore further the relationship between stress-induced cognitive intrusions and insomnia on depression-risk. Daily diary designs supplemented with wearable technology would offer a more intimate look at the dynamics between nightly inability to sleep and nocturnal cognitive arousal, while leveraging the benefits of both subjective and objective estimates of sleep parameters. Although present study shows that premorbid insomnia symptoms and cognitive intrusions increase depression-risk in response to stress, daily diary studies will offer more nuanced insights into the evolution of the reciprocal dynamics between insomnia symptoms and perseverations over time that give rise to depression. It is also important for laboratory studies to examine more closely the causal mechanisms between nocturnal cognitive arousal and insomnia on mood. For instance, laboratory experiments utilizing neutrally-valenced wakefulness-induction to prolong sleep latency (such as circadian phase advancement) can be used to compare high vs low trait perseverators on nocturnal cognitive arousal and next-day affect.

## Supporting information

S1 Dataset This data set is an SPSS file used for data analysis for the present study. (ZIP)

S1 Table This table represents a variable key to accompany SPSS data set, describing variables used in analysis for the present study. (XLSX)
